# Endophytic bacteria *Priestia megaterium* 170T-4 improves soybean salt tolerance through regulation of ion homeostasis and phytohormone signaling pathways

**DOI:** 10.3389/fmicb.2025.1676456

**Published:** 2025-09-25

**Authors:** Shutian Hua, Ruiyao Liu, Zhe Li, Han Zheng, Yanfen Zheng, Youqiang Wang, Cheng-Sheng Zhang, Ziyan Wang, Mingguo Jiang

**Affiliations:** ^1^Guangxi Key Laboratory for Polysaccharide Materials and Modifications, School of Marine Sciences and Biotechnology, Guangxi Minzu University, Nanning, China; ^2^Marine Agriculture Research Center, Tobacco Research Institute of Chinese Academy of Agricultural Sciences, Qingdao, China; ^3^National Center of Technology Innovation for Comprehensive Utilization of Saline-Alkali Land, Dongying, China

**Keywords:** salt stress, soybean, endophytic bacteria, *Priestia megaterium* 170T-4, plant growth-promoting rhizobacteria

## Abstract

Salt stress in coastal saline-alkali soils impairs plant survival and growth. Plant growth-promoting rhizosphere bacteria (PGPR) and endophytic bacteria can enhance salinity tolerance via stable host associations. This study used culture-based and transcriptomic methods to investigate culturable endophytic bacteria in soybean and their salt-tolerance mechanisms. A total of 154 strains were isolated from the roots of 10 soybean varieties cultivated in coastal saline-alkali soil, spanning 4 phyla, 35 genera, and 76 species. *Microbacterium phyllosphaerae* and *Priestia megaterium* were identified as dominant species, from which two representative strains were selected to assess their growth-promoting effects under salt stress. Strain 170T-4 was identified as *P. megaterium* via multilocus sequence analysis and showed high salt tolerance, growing in up to 6% NaCl. Pot experiments showed that strain 170T-4 significantly improved plant height, root elongation, Na^+^/K^+^ homeostasis, proline, and chlorophyll content. Transcriptome profiling and RT-qPCR revealed that strain 170T-4 regulates K^+^ transport-related genes (*GORK* and *SKOR*), ethylene signaling related genes (*PTI5*, *EIN3*, and *ERF1*), and the allene oxide cyclase gene (*AOC*). Overall, strain 170T-4 improved soybean growth under salt stress by modulating ion transport, osmotic responses, and hormone signaling, showing strong potential as a microbial inoculant for saline-alkali soils.

## 1 Introduction

Salinity has been identified as one of the major abiotic stresses threatening global agricultural production (Singh and Gautam, 2013), with severe implications for sustainable agriculture and global food security. High salt environments disrupt plant ion homeostasis, inhibit photosynthesis, and reduce seed germination and crop yield (Liu et al., 2025). Soil salinity problems have been ameliorated to some extent by current mainstream improvement strategies such as breeding, genetic improvement, crop rotation and application of exogenous substances (Dong et al., 2024). However, their large-scale implementation remains constrained by substantial economic investments and time-intensive requirements.

In recent years, plant growth-promoting rhizobacteria (PGPR) have garnered significant attention in stress biology research due to their multifaceted bio-enhancing capacities (Lebeis, 2015; Bhat et al., 2020). Accumulating evidence demonstrates that PGPR effectively enhance plant salt tolerance through synergistic mechanisms (Ling et al., 2022; Yu et al., 2021). PGPR secrete plant hormones such as indole-3-acetic acid (IAA) (Bartholomew Saanu et al., 2021; Olanrewaju et al., 2017), which promote root growth and consequently improve soybean salt tolerance (Lu et al., 2024). And through the production of 1-aminocyclopropane-1-carboxylic acid (ACC) deaminase, PGPR catabolize ACC into ammonia and α-Ketobutyric acid, thereby attenuating ethylene-induced growth inhibition (Abdul et al., 2024). Additionally, PGPR contribute to cellular osmotic balance by synthesizing osmoprotectants such as proline and soluble sugars (Naveena and Chitra, 2024; Yang and Xinwei, 2025). They also mitigate oxidative stress damage by enhancing the activity of antioxidant enzymes, including superoxide dismutase (SOD) and peroxidase (POD), which eliminate excessive reactive oxygen species (ROS) (Abdelkrim et al., 2018; Han et al., 2014). Furthermore, PGPR participate in ionic homeostasis maintenance by modulating ion transport systems to facilitate potassium (K^+^) uptake while restricting sodium (Na^+^) accumulation (Chenghua et al., 2024; Yue et al., 2023).

Notably, endophytic bacteria colonizing the internal tissues of plants often demonstrate greater effectiveness in enhancing host stress tolerance than rhizosphere bacteria (Compant et al., 2005). Their unique niche provides physical protection from abiotic stresses such as salinity and enables closer physiological integration with the host (Hardoim et al., 2015). This proximity facilitates more efficient modulation of plant responses-including phytohormone signaling and the regulation of stress-related genes-promoting more direct and sustained beneficial effects (Rolli et al., 2015). Therefore, the exploitation of endophytes represents a promising strategy for developing microbial agents to mitigate salt stress.

*Priestia* spp. represents a typical PGPR (Hwang et al., 2022). A research has demonstrated that *P. endophytica* can promote plant growth under salt stress through the processes of nitrogen assimilation and secondary metabolite production (Sharma et al., 2022). However, no studies have confirmed the efficacy of *Priestia* spp. in alleviating salt stress in soybean (*Glycine max*). Soybean, as the fourth most cultivated global food crop, plays a critical role in maintaining food security (Gabriela Eugenia et al., 2025) and advancing sustainable agriculture. In the context of the concurrent constraints on arable land resources and increasing demand, the development and utilization of saline and alkaline land has emerged as a pivotal strategy for augmenting soybean production capacity (Ci et al., 2023). However, as a typical salt-sensitive crop, soybean demonstrates limited agricultural productivity in saline environments (Fazl et al., 2024). Therefore, it is extremely important to improve soybean salt tolerance. As an environmentally friendly and sustainable agricultural strategy, the application of PGPR can enhance crop performance under saline conditions by inducing host plant tolerance mechanisms (Bharti et al., 2016). Therefore, identifying effective PGPR strains and elucidating their mode of action are critical for improving soybean salt tolerance, crop quality, and overall agricultural sustainability (Wang et al., 2023).

In this study, we isolated endophytic bacteria from the root systems of soybean cultivars and obtained a strain of *P. megaterium* 170T-4 with growth-promoting properties. Through pot experiments, we investigated the mechanism underlying strain 170T-4-mediated salt stress alleviation in soybean through integrated analysis of plant growth phenotypes, physiological and biochemical indices, and molecular regulatory networks. These findings will lay a foundation for the targeted development of salt-tolerant microbial agents.

## 2 Materials and methods

### 2.1 Isolation and identification of endophytic bacteria from soybean roots

Ten different soybean varieties (Supplementary Table S1) were grown in pots with a diameter of 9 cm, each containing 200 g of saline soil (soil electrical conductivity of ~600 μs/cm and pH 8.7) collected from Dongying, Shandong Province (37°17′30″N, 118°37′56″E, North China). On day 21 after sowing, the roots of these soybean cultivars were collected, surface-sterilized with 75% ethanol for 5 min, washed with sterile water. Then the roots were transferred to a sterile mortar containing 3 mL of sterile water and crushed (Hallmann et al., 1997). The effectiveness of the disinfection process has been demonstrated through repeated experiments (Supplementary Figure S1). A 200 μL aliquot of the diluent (10^−1^, 10^−2^, 10^−3^) was spread onto TSA and R_2_A plates, respectively, and incubated in an incubator at 28 °C for 2–3 days. Single colonies with different morphologies were selected and transferred to fresh TSA and R_2_A plates. After purification, the strains were preserved in glycerol and stored at −80 °C for further study.

The DNA of soybean endophytic bacteria was extracted using the boil-cracking method, then amplified and sequenced using 16S rRNA universal primers. The primer sequences used were 27F (5′-AGAGTTTGATCCTGGCTCAG-3′) and 1492R (5′-GGTTACCTTGTTACGACTT-3′) (Weisburg et al., 1991). PCR amplification was performed in a 25 μL mixture containing 12.5 μL of 2 × Taq Master Mix (Vazyme, China), 1 μL of each primer, ddH_2_O 8.5 μL and 2 μL genomic DNA. PCR amplification was performed as follows: initial denaturation at 95 °C for 5 min; 30 cycles of denaturation at 95 °C for 1 min, annealing at 55 °C for 1 min, and extension at 72 °C for 1.5 min; with a final extension at 72 °C for 10 min (Sui et al., 2024). The amplified products were sequenced by Liuhe BGI Co., Ltd. (Beijing, China). And the sequences were submitted to EzBioCloud (https://www.ezbiocloud.net/) for 16S rRNA sequence alignment to determine the species of the target isolates. MEGA 11.0 software was used to perform CLUSTALX multiple sequence alignment, and the Neighbor-Joining method was employed for phylogenetic analysis. The target strains were selected from the dominant genera with greater enrichment, and the subsequent pot experiment was carried out.

To determine the classification of the 170T-4 strain, a phylogenetic tree was constructed based on multilocus sequences. The housekeeping genes *recA*, *rpoD*, and *gyrB* of 170T-4 were amplified and sequenced (the primer sequences are listed in Supplementary Table S2). The PCR amplification procedure was consistent with the 16S rRNA gene amplification procedure. Multiple sequence alignment was performed using MUSCLE (Edgar, 2004) within the MEGA11 software package. A concatenated phylogenetic tree was constructed using the Neighbor-Joining method and analyzed using 1,000 bootstrap replicates.

### 2.2 Seed germination, bacterial culture and salt treatment

The pot experiment was carried out with *Glycine max* “Zhonghuang 13.” Vermiculite was used as the cultivation medium, with 5 soybean seeds sown in each pot with a diameter of 9 cm. To ensure the uniformity of all test pots, seeds germinate were performed under controlled conditions. After germination, only healthy, uniform in shape were selected and transplanted into the experimental pots. The pots were cultured in a 14-h light/10-h dark incubator and watered regularly. Four treatment groups were established: the control group (MOCK), salt stress group (Salt), inoculation group (X), and the salt stress inoculation group (X + Salt). X represents various bacterial strains. Each treatment had 6 replicates.

The isolated and purified bacterial strains were inoculated into nutrient broth (NB) medium. After culturing under shaking at 28 °C and 180 rpm for 24 h, the bacterial cells were collected and resuspended in sterile water (OD_600_ = 0.3, equivalent to 3 × 10^7^ CFU/mL). Following seed germination, 20 mL of bacterial suspension was inoculated into the inoculation group and the salt-stressed inoculation group three times. The plants were treated twice, each time with 20 mL of 400 mmol/L NaCl per pot, using a graduated centrifuge tube for accurate measurement (the control group was treated with the same amount of distilled water). And 14 days after the final treatment, soybean leaves and roots from different treatment groups were collected, frozen in liquid nitrogen, and stored at −80 °C for physiological analysis and RNA extraction. The growth indicators of soybean seedlings, including fresh weight, plant height and root length, were measured, and the growth-promoting bacteria were further screened.

### 2.3 Determination of salt-tolerant of strains

A salt concentration gradient ranging from 0–8% (w/v) was established. The bacterial solution of strain 170T-4 was inoculated into NB medium with different salt concentrations at a 2% inoculating rate and incubated at 28 °C. After 60 h of cultivation, the optical density at 600 nm (OD_600_) was measured using a microplate reader. Three replicates were performed for each condition.

### 2.4 Determination of plant physiological parameters

#### 2.4.1 Determination of Na^+^ and K^+^ content

After 14 days of salt stress treatment, the soybean root samples were thoroughly washed and dried to a constant weight. The dried samples were weighed, cut into small pieces, and transferred to digestion tubes. Concentrated nitric acid was added according to the sample weight (0.025 g/mL). The samples were then heated in a metal bath at 110 °C overnight. After complete digestion, the solutions were transferred to centrifuge tubes and the volume were adjusted to 4 mL with ddH_2_O. The digested solutions were filtered through 0.22 μm filter membranes and diluted 20-fold. Then the contents of Na^+^ and K^+^ were determined using an Optima 8000 plasma emission spectrometer (ICP).

#### 2.4.2 Proline content determination

The content of proline in leaves was determined using sulfosalicylic acid method (Chernysheva et al., 2024). Soybean leaves (0.1 g) were flash-frozen in liquid nitrogen and ground into powder, then homogenized in 3 mL 3% sulfosalicylic acid solution. The mixture was extracted in boiling water for 15 min, immediately cooled on ice for 2 min, and centrifuged at 4,000 rpm for 10 min. The resulting supernatant was collected for analysis. For the sample supernatant and standard solution, 250 μL of each was mixed with 250 μL of glacial acetic acid and 250 μL of ninhydrin reagent, followed by incubation in a 100 °C water bath for 30 min. After cooling to room temperature, 500 μL of toluene was added to each tube and mixed thoroughly. Following phase separation, 200 μL of the toluene layer was transferred to a microplate, and the absorbance was measured at 520 nm. A standard curve was then generated, and the content of proline was calculated according to the standard curve.

#### 2.4.3 Determination of photosynthetic pigment content

Chlorophyll content was determined by spectrophotometry (Dudek et al., 2013; Demidov and Baulin, 1995). Soybean leaves (0.1 g) were ground into powder in liquid nitrogen, extracted with 20 mL 95% ethanol, centrifuged at 4,000 rpm for 10 min to obtain supernatant. The absorbance value was measured at 440 nm, 649 nm and 665 nm, and the chlorophyll content was calculated according to the following formula. Chlorophyll a concentration (mg/L) = 12.71 × A_665_–2.59 × A_649_. Chlorophyll b concentration (mg/L) = 22.88 × A_649_–4.67 × A_665_.

#### 2.4.4 Determination of antioxidant enzyme activity

Soybean leaves (0.1 g) were ground into powder in liquid nitrogen, followed by homogenization in 1 mL of extraction buffer on ice. After centrifugation at 4,000 rpm at 4 °C for 10 min, the supernatant was collected and kept on ice for subsequent analysis. The activity of superoxide dismutase (SOD) was measured using the nitroblue tetrazolium (NBT) photochemical reduction method (Cao et al., 2022). Peroxidase (POD) activity was assessed via the guaiacol oxidation method (Cao et al., 2022), and catalase (CAT) activity was determined by the hydrogen peroxide redox method (Cao et al., 2022). These kits were purchased from Solarbio Science & Technology Co., Ltd. (Beijing, China).

#### 2.4.5 Determination of malondialdehyde content

The content of malondialdehyde (MDA) in leaves was determined by thiobarbituric Acid (TBA) reactivity assay (Kovalikova et al., 2020). Soybean leaves (0.1 g) were ground into powder in liquid nitrogen, and mixed with 3 mL of 10% trichloroacetic acid (TCA). The homogenate was centrifuged at 4,000 rpm for 10 min, and the supernatant was collected. An equal volume of 0.6% TBA was added to the supernatant, then the mixture was heated at 100 °C for 15 min, followed by rapid cooling in an ice bath. After centrifugation at 10,000 rpm for 15 min, the absorbance value of the supernatant was measured at 450 nm, 532 nm and 600 nm by ultraviolet spectrophotometer, and the MDA content was calculated according to the following formula. MDA concentration (μmol/L) = 6.45 × (A_532_-A_600_)–0.56 × A_450_.

### 2.5 Transcriptome sequencing and analysis

Root samples from four treatment groups were collected, rapidly frozen in liquid nitrogen, and stored at −80 °C. Three biological replicates were performed for each treatment group. Total RNA extraction, reverse transcription, cDNA library construction, and sequencing were carried out according to the method described by Sui et al. (2024). To obtain high quality clean reads, raw RNA-seq data were processed with Fastp to remove adapter sequences, low quality read segments, sequences with ambiguous base information and short reads. The clean reads were then mapped to the *Glycine_max*_v2.1 reference genome (http://plants.ensembl.org/Glycine_max/Info/Index) with HiSat2 to generate read counts. The transcript abundance for each sample was quantified as raw read counts using RSEM (RNA-Seq by Expectation–Maximization). These raw count matrices were then used as the direct input for differential expression analysis with DESeq2, which applies its internal normalization procedure (the “median of ratios” method) to account for differences in library size and RNA composition. For the visualization and comparison of gene expression levels across samples, we utilized the TPM (Transcripts Per Million) values. Differentially expressed genes (DEGs) were screened using DESeq2, with the criteria of an adjusted *p*-value (*P*-adjust) < 0.05 (Benjamini-Hochberg method) and |log_2_FC| ≥ 1. Functional annotation was performed through Gene Ontology (GO) and Kyoto Encyclopedia of Genes and Genomes (KEGG) enrichment analyses with Goatools and KOBAS (Wang et al., 2024). The raw sequence data have been deposited in the Genome Sequence Archive of China National Center for Bioinformation (CNCB) under the accession number CRA029786. These data are publicly accessible at https://ngdc.cncb.ac.cn.

### 2.6 RT-qPCR analysis

Total RNA was extracted from the samples using the RNA Kit reagent (YEASEN, Shanghai, China) according to the manufacturer’s instructions. First-strand cDNA was synthesized via a HiScript II 1st Strand cDNA Synthesis Kit (Vazyme Biotech, Nanjing, China) according to the manufacturer’s protocol. RT-qPCR was performed using 2 × Q5 SYBR qPCR Master Mix (Universal) (Tolo Biotech Co., Ltd., China). *GmCYP2* was used as a housekeeping gene. Differentially expressed genes were selected for RT-qPCR validation included *GLYMA.12G076800* (*CNGC*), *GLYMA.17G118300* (*AKT*, *KAT*, *GORK*, and *SKOR*), *GLYMA.19G043000* (*PRODH*, *fadM*, and *putB*), *GLYMA.04G020800* (*P4HA*), *GLYMA.02G016100* (*EREBP*) and *GLYMA.18G280900* (*AOC*). And the specific primers designed with Primer Premier 5.0 (Supplementary Table S2). Each sample was analyzed in three biological replicates, and relative expression levels were obtained using the 2^−△△Ct^ method (Sui et al., 2024).

## 3 Results

### 3.1 Isolation and screening of endophytic bacteria from soybean roots

**Figure 1:**
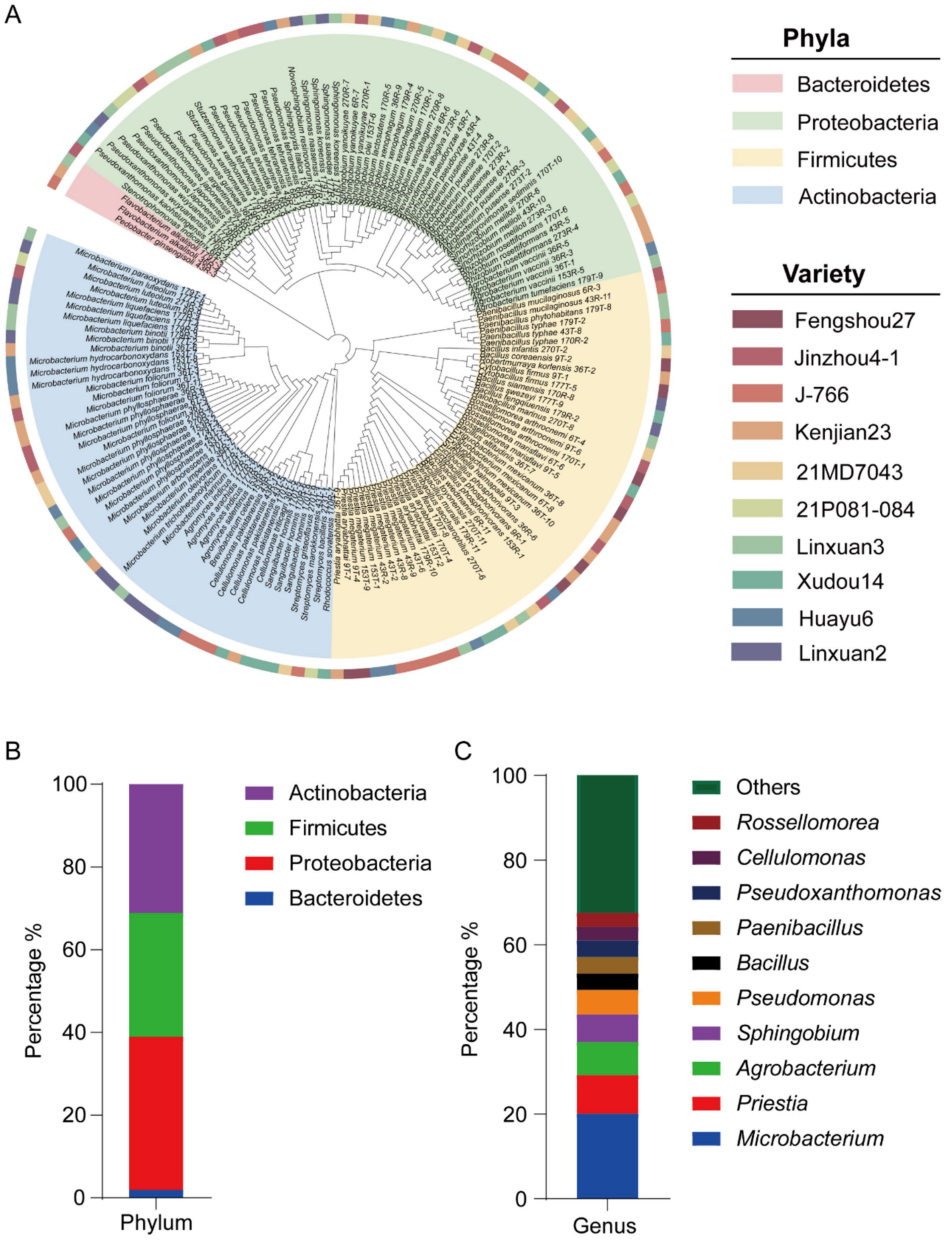
Isolation and screening results of 154 strains of endophytic bacteria from soybean roots. **(A)** Evolutionary tree based on 16S rRNA gene sequences of 154 strains. **(B)** Endophytic bacteria at the phylum level. **(C)** Endophytic bacteria at the genus level.

A total of 154 endophytic bacteria were isolated from the roots of 10 soybean cultivars grown under uniform soil conditions (Figure 1A; Supplementary Table S1). According to 16S rRNA gene similarity analysis, these bacterial strains belonged to 4 phyla, 6 classes, 14 orders, 17 families, 35 genera and 76 species. The four phyla were Proteobacteria (57 strains), Actinomyces (48 strains), Firmicutes (46 strains), and Bacteroidetes (3 strains) (Figure 1B). At the genus level, *Microbacterium*, *Priestia*, *Agrobacterium*, *Sphingobium* and *Pseudomonas* were the most five abundant genera, accounting for 20.1, 9.1, 7.1, 6.5, and 5.8% of the total isolates, respectively (Figure 1C). Among them, *M. phyllosphaerae* and *P. megaterium* were the most abundant species, accounting for 29.0 and 57.1% among their respective genera.

### 3.2 Effects of target strains on soybean seedling biomass under salt stress

**Figure 2:**
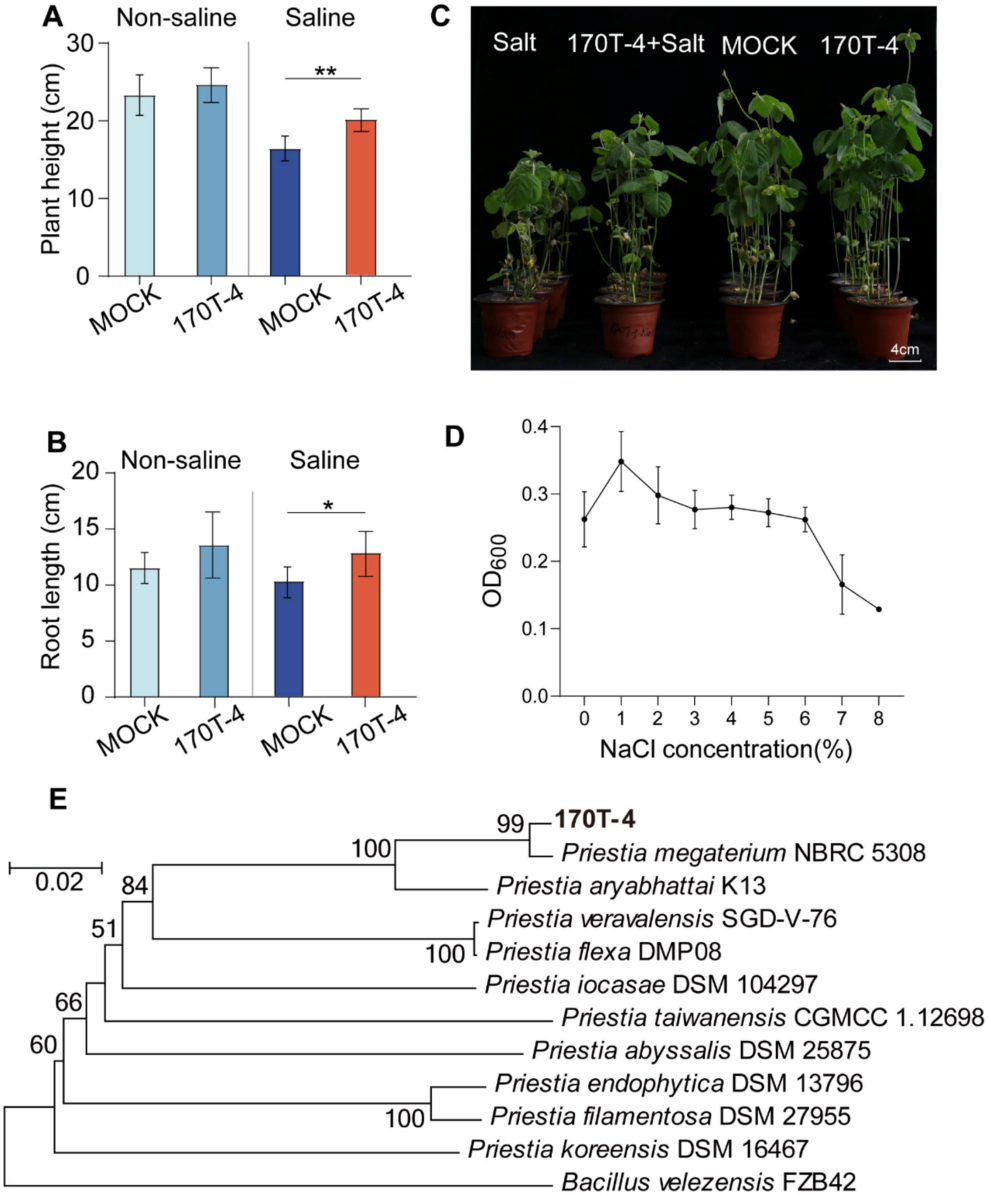
*P. megaterium* 170T-4 alleviated the adverse effects of salt stress on soybean seedlings. Plant height **(A)** and root length **(B)** of soybean under different treatments. **(C)** Growth of simulated-inoculated and inoculated soybeans under normal and salt stress conditions. **(D)** Evaluation of salt tolerance of strain 170T-4. Asterisk indicates that the *p* value of the two-tailed student’s *t*-test is less than 0.05 (*p < 0.05, **p < 0.01). **(E)** Phylogenetic tree based on concatenated sequences of *recA*, *rpoD*, and *gyrB* housekeeping genes.

Two representative strains *M. phyllosphaerae* 153 T-5 and *P. megaterium* 170T-4 were selected for pot experiments to evaluate the effects of these strains on soybean salt tolerance and growth performance under salt stress. The results showed that inoculation with *M. phyllosphaerae* 153 T-5 exhibited no significant effects on plant height and root length of soybean (Supplementary Figure S3). For *P. megaterium* 170T-4, no statistically significant differences (*p* > 0.05) were observed in either plant height or root length between 170T-4 inoculated and mock-inoculated soybean plants under non-salt stress conditions. However, under salt stress conditions, plants inoculated with strain 170T-4 exhibited significant growth promotion, with a 22.18% increase in plant height (*p* < 0.01) and a 24.80% enhancement in root length (*p* < 0.05) compared to MOCK (Figures 2A,B). These results suggest that plant growth enhancement induced by strain 170T-4 was primarily specific to salt stress condition (Figure 2C). Subsequently, the salt tolerance of strain 170T-4 was evaluated by determining its growth under different salt concentration. The results showed that 1% NaCl was the optimal concentration for the growth of strain 170T-4, and the strain retained certain viability even at a NaCl concentration of 6% (Figure 2D). To further identify the classification of strain 170T-4, a phylogenetic tree was constructed based on a multilocus sequence analysis of *recA*, *rpoD*, and *gyrB*. This analysis confirmed that strain 170T-4 belongs to the species *P. megaterium* (Figure 2E).

### 3.3 Effects of strain 170T-4 on physiological parameters of soybean

**Figure 3:**
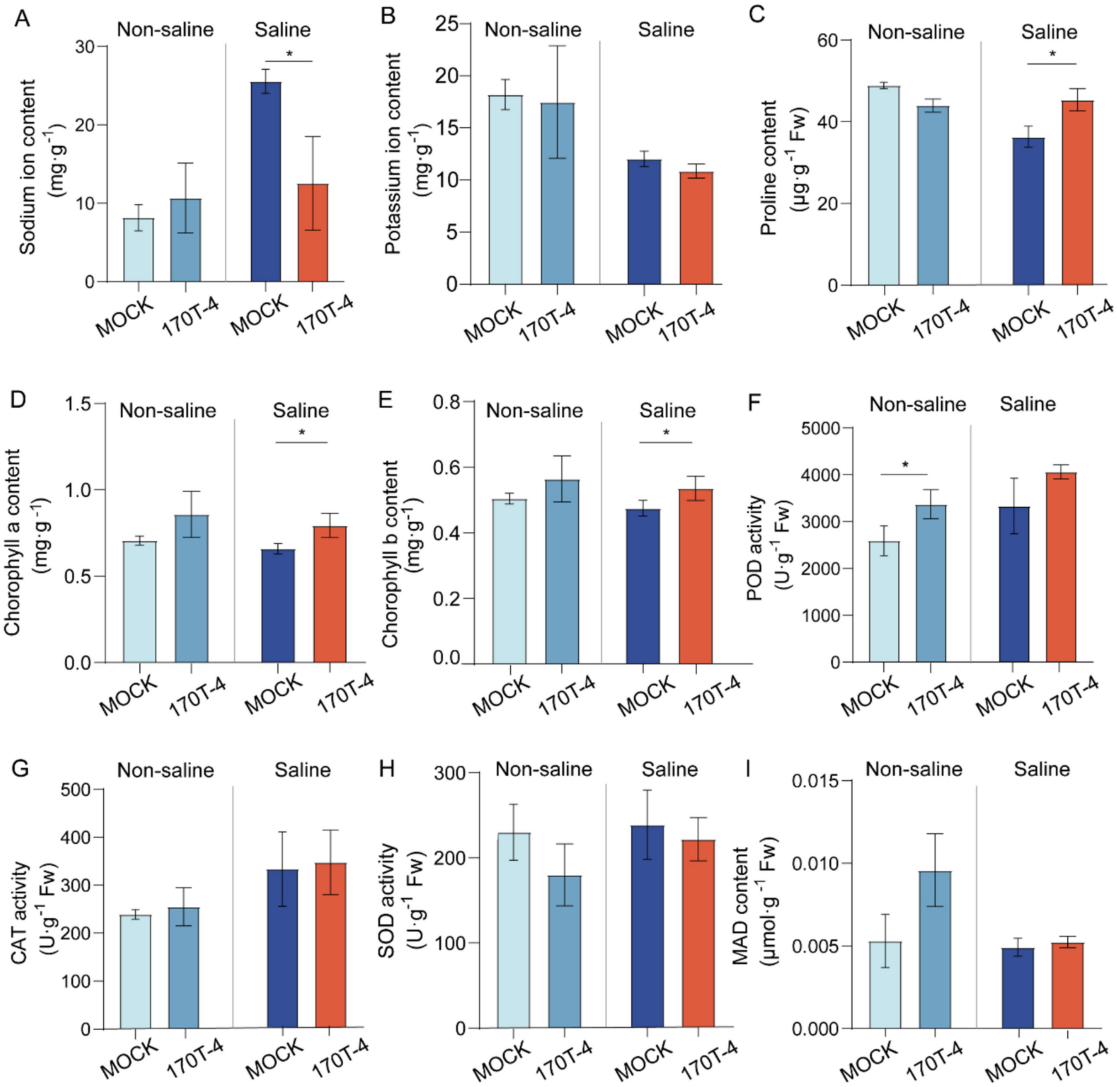
Effects of strain 170T-4 on physiological parameters of soybean. Effects of inoculated strain 170T-4 on contents of Na^+^ **(A)**, K^+^ **(B)**, proline **(C)**, chlorophyll a **(D)**, chlorophyll b **(E)**, activities of antioxidant enzymes including POD **(F)**, CAT **(G)**, SOD **(H)** and MDA contents **(I)** in soybean under normal and salt stress conditions. Asterisk indicates that the *p* value of the two-tailed student’s *t*-test is less than 0.05 (*p < 0.05, **p < 0.01).

To identify the patterns of strain 170T-4 in enhancing soybean growth under salt stress, we tested the Na^+^ content and other biochemical parameters of soybean. The results showed that under salt treatment conditions, the Na^+^ content in the root of soybean inoculated with 170T-4 significantly decreased by 50.99% compared to MOCK (Figure 3A). Additionally, under salt stress conditions, the 170T-4 inoculation group showed significant increases compared to MOCK, with a 24.89% higher proline content (Figure 3C), along with 20.33 and 12.75% increases in chlorophyll a and b contents, respectively (Figures 3D,E). However, under salt stress, there were no significant changes in K^+^ content (Figure 3B), POD, CAT, SOD activities (Figures 3F–H), or MDA level (Figure 3I) between 170T-4-inoculated soybean and mock-inoculated soybean plants. Under non-salt stress conditions, inoculation with 170T-4 only significantly enhanced POD activity of soybean, while no significant differences were observed in other measured indices compared with the mock-inoculated control group (Figure 3F).

### 3.4 Transcriptome analysis of soybean after 170T-4 inoculation

**Figure 4:**
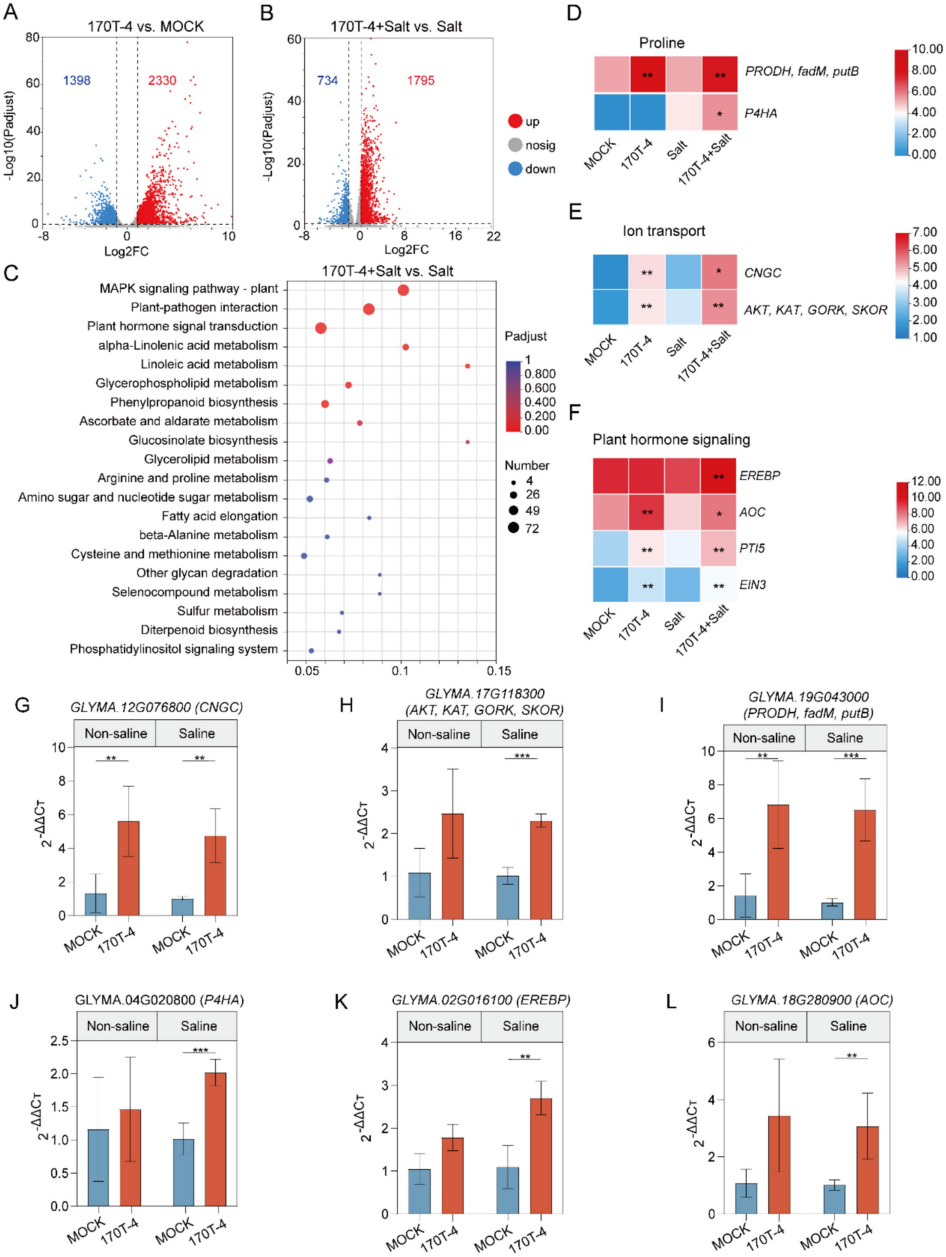
Transcriptional responses of soybean to 170T-4 inoculation. **(A)** Volcano plot: number of differentially expressed genes (DEGs) in soybean under mock-inoculated or inoculated conditions without salinity. **(B)** Volcano plot: number of DEGs in mock-inoculated or inoculated soybean plants under salt stress. **(C)** KEGG enrichment analysis of response of 170T-4 inoculated soybean to salt stress. **(D–F)** Differential gene expression related to proline, ion transport and plant hormone conduction changed significantly. **(G–L)** The RT-qPCR verification of DEGs related to salt stress.

To further explore the molecular mechanisms of the response of soybeans to salt stress after inoculation with *P. megaterium* 170T-4, we performed transcriptomic analysis of soybean plants 14 days after the last salt treatment using RNA sequencing (RNA-seq). Mock-inoculated and 170T-4 inoculated plants were compared under salt treatment and control conditions, with three biological replicates for each group. The Clean Data of all samples reached more than 6.46 Gb, and the percentage of Q30 bases was above 97.24% (Supplementary Table S3). Over 95% of the clean reads were mapped to the soybean reference genome *Glycine_max*_v2.1 (http://plants.ensembl.org/Glycine_max/Info/Index). Under non-salt stress conditions, 2,230 genes in the 170T-4 inoculated group were significantly upregulated, while 1,398 genes were downregulated compared to MOCK (Figure 4A). Under salt stress, 1,795 genes in the 170T-4 inoculated group were upregulated (Supplementary Table S4), and 734 genes were downregulated (Figure 4B). In conclusion, inoculation with 170T-4 significantly influenced the soybean response to salt stress. KEGG enrichment analysis of the upregulated DEGs revealed the top 20 most enriched pathways (Figure 4C). The upregulation of DEGs induced by salt stress was significantly enriched in pathways such as the MAPK signaling pathway, plant-pathogen interaction, plant hormone signal transduction, linolenic acid metabolism, linoleic acid metabolism, and phenylpropanoid biosynthesis.

**Figure 5:**
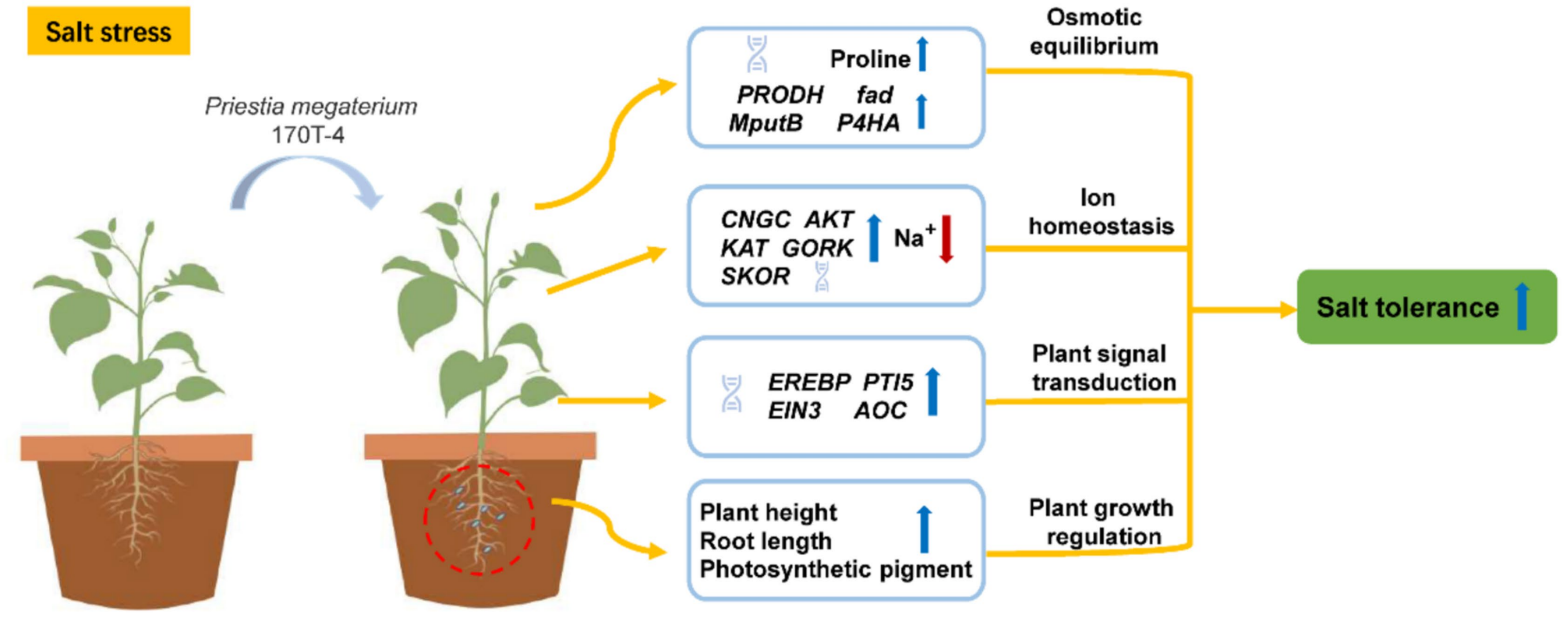
Model diagram of strain *P. megaterium* 170T-4 alleviating salt stress in soybean.

Based on the results of physiological and biochemical indexes, special attention was given to the KEGG Orthology (KO) functional categories directly associated with soybean growth, stress resistance, and plant growth promotion under salt stress, including osmoregulation, ion transport, and plant hormone signal transduction. In terms of osmoregulation, inoculation with *P. megaterium* 170T-4 significantly increased the expression quantity of *PRODH*, *fadM* and *putB* under both salt and non-salt conditions (Figure 4D). Under salt stress, the transcription level of *P4HA* also significantly increased, suggesting that 170T-4 regulates the metabolic pathway of proline to maintain osmotic balance and help plants maintain normal physiological function and growth under high salt environment. In response to ion transport under salt stress, the expressions of *CNGC*, *AKT*, *KAT*, *GORK*, and *SKOR* were significantly upregulated following inoculation with 170T-4 (Figure 4E). This helps maintain the balance of Na^+^ and K^+^ within cells, in addition to facilitating Ca^2+^ regulation, thus alleviating the detrimental effects of salt stress on plants. In plant hormone signal, the expression levels of *EREBP*, *AOC*, *PTI5*, and *EIN3* were significantly increased after 170T-4 inoculation under salt stress (Figure 4F). By regulating the ethylene signal pathway and the expression of *AOC* (allene oxide cyclase), a key enzyme in JA biosynthesis, soybean growth and development and adaptation to environmental stress were promoted. The expression patterns of differentially expressed genes related to salt stress were validated by RT-qPCR. The genes involved in ion transport (*CNGC*, *AKT*, *KAT*, and *GORK*), proline metabolism (*PRODH*, *fadM*, *putB*, and *P4HA*), and plant hormone signal transduction (*EREBP* and *AOC*) were all significantly upregulated. The RT-qPCR results generally consistent with the RNA-seq data (Figures 4G–L), confirming the reliability of the RNA-seq results. Overall, 170T-4 significantly improved soybean growth and physiological traits under salt stress by regulating ion transport, osmotic protective substances, and hormone signaling (Figure 5).

## 4 Discussion

PGPR are microorganisms living in plant rhizosphere soil, which enhance plant growth and development through various mechanisms, such as phosphorus dissolution, nitrogen fixation, plant hormone secretion, and improving plant survival under adverse conditions (Yang et al., 2023; Kamogelo et al., 2025). As native inhabitants of plant tissues, endophytic bacteria develop stable mutualistic relationships with hosts, offering more persistent benefits than PGPR from other sources. Consequently, the present study targets soybean-derived endophytic bacteria, aiming to systematically identify strains with salt tolerance-promoting potential and investigate the underlying mechanism. Specifically, 154 bacterial strains (belonging to 35 genera) were isolated from the roots of 10 soybean cultivars. The significant diversity of endophytes observed under uniform soil conditions suggests that host-specific factors primarily drive this variability (Liu et al., 2019). The genetic background of different soybean cultivars likely plays a significant role, with each variety potentially recruiting distinct bacterial partners through variations in root exudate profiles (Yuliya et al., 2017). This study used TSA and R_2_A for the combined isolation and culture of eutrophic and oligotrophic endophytic bacteria. This method improved the diversity of culturable microorganisms to some extent. Since rhizobium-specific culture media were not used for bacterial isolation in this study, rhizobia were not obtained. In future research, a combination of diverse culture media will be employed to isolate a more comprehensive and diverse range of microbial communities. In addition, we recognize that using fluorescent labeling of the bacterium and analyzing its colonization patterns under a confocal microscope is an excellent method for confirming its endophytic nature. This approach will be integrated into future studies. Pot experiment demonstrated that only *P. megaterium* 170T-4 enhanced soybean growth performance under salt stress conditions. And in the salt-tolerant growth test of bacteria, this strain showed excellent salt adaptation capacity (surviving under the NaCl concentrations up to 6%). Therefore, *P. megaterium* 170T-4 was selected for further investigation. Pot experiment and measurements of salt tolerance-related indicators were conducted to verify the ability of strain 170T-4 to enhance salt stress resistance in soybean and to elucidate its underlying mechanisms. Nevertheless, as we did not conduct pot experiments on all isolated strains, the possibility cannot be excluded that strains capable of enhancing soybean salt tolerance exist among the others.

Studies have shown that PGPR can promote the synthesis of osmotic protective substances in plants, such as proline and betaine (Giannelli et al., 2023). These osmoprotective substances can stabilize cell structure, maintain osmotic pressure balance within cells, and prevent cells from dehydration due to salt stress (Ashraf and Foolad, 2007). In this study, under salt stress, the proline content of soybean leaves inoculated with 170T-4 showed a 24.89% increase compared to the simulated-inoculated soybean plants. Based on KEGG metabolic pathway enrichment analysis, it is hypothesized that 170T-4 may improve the ability of plants to regulate osmosis under stressful conditions by regulating the expression of genes related to proline metabolism. A previous study showed that, *P. megaterium* helps plants produce stronger antioxidant enzyme activity, reduces oxidative damage to plant cells, and improves plant stress resistance (Shahid et al., 2022). However, in our study, strain 170T-4 significantly increased the content of chlorophyll in soybean leaves, but did not significantly induce the change of antioxidant enzyme activity in soybean under salt stress. This indicated that strain 170T-4 alleviates salt stress in soybean through mechanisms independent of antioxidant pathways.

Regulation of Na^+^ and K^+^ transport in plants is a key mechanism by which PGPR alleviates salt stress (Aasia et al., 2024). *Bacillus subtilis* GB03, isolated from soil, reduces Na^+^ accumulation and increases the K^+^/Na^+^ ratio under high salt conditions, helping wheat resist salt-alkali toxicity (Zhang et al., 2014). In this study, *P. megaterium* 170T-4 significantly reduced Na^+^ content in soybean roots under salt stress. High Na^+^ levels in the plasma membrane are balanced by several mechanisms, including K^+^ absorption through potassium channels, salt rejection mechanisms, and Na^+^ sequestration in vacuoles (Malakar and Chattopadhyay, 2021; Joshi et al., 2024). Further transcriptome analysis of soybean root systems suggested that strain 170T-4 may regulate the expression of the GORK and SKOR genes, which encode outward-rectifying K^+^ channels. It is hypothesized that this regulation may help to maintain Na^+^/K^+^ homeostasis within cells, thereby reducing salt stress damage in soybeans. Further functional verification could be achieved through experiments such as gene knockout design in future studies.

As an important member of the PGPR, *P. megaterium* enhances host resistance by modulating the phytohormone signaling network (Waadt et al., 2022; Naeem, 2025). For instance, *P. megaterium* promotes soybean growth by increasing the levels of phytohormones such as abscisic acid (ABA) and gibberellins (GA3), while also exhibiting tolerance to oxidative stress (Almiron et al., 2025). The activation of ethylene and jasmonic acid (JA) signaling induces defense gene expression, improving resistance to pathogens and abiotic stresses like drought and salt stress (Song and Wu, 2023). In this study, KEGG metabolic pathway enrichment analysis revealed two key regulatory pathways: the MAPK signaling pathway and the phytohormone signaling pathway. Specifically, after inoculation with 170T-4 under salt stress, the core transcription factors of the ethylene signaling pathway (*PTI5, EIN3, ERF1*) were activated through the MAPK cascade reaction, which helped plants to resist salt stress (Bukhat et al., 2020; Merchante et al., 2013). Notably, after inoculating 170T-4 under salt stress, gene expression of AOC (allene oxide cyclase), a key enzyme in linolenic acid metabolism, was significantly upregulated. As a rate-limiting enzyme in JA biosynthesis, AOC activation improves JA production, boosting defense gene expression and stress resistance. This mechanism enhances plant tolerance to both biotic stresses (e.g., pathogens) and abiotic stresses (e.g., drought, salt) (Hewedy et al., 2023; Kumar et al., 2024). The results speculated a synergistic mechanism: 170T-4 enhances stress responses via ethylene signaling pathway and promotes growth through the linolenic acid metabolism-JA synthesis pathway, improving soybean adaptability under salt stress.

## 5 Conclusion

In conclusion, this study isolated 154 endophytic bacterial strains from 10 soybean cultivars. Based on its proven ability to improve salt tolerance in soybean, *P. megaterium* 170T-4 was selected for future research from the dominant genus. Pot experiments demonstrated that 170T-4 significantly improved soybean growth parameters (plant height and root length), maintained Na^+^/K^+^ homeostasis, and increased proline and chlorophyll content under salt stress. Transcriptomic analysis and RT-qPCR uncovered the molecular mechanisms by which 170T-4 enhances salt tolerance, primarily through the regulation of ion transport, osmotic stress responses, and plant hormone signaling pathways. These findings provide mechanistic insights into endophyte-mediated salt tolerance in soybean and support the potential application of *P. megaterium* 170T-4 as a microbial inoculant for improving soybean productivity in saline soils.

## Data Availability

The datasets presented in this study can be found in online repositories. The names of the repository/repositories and accession number(s) can be found in the article/Supplementary material.
