# Supplementary figures and images for: Endophytic bacteria Priestia megaterium 170T-4 improves soybean salt tolerance through regulation of ion homeostasis and phytohormone signaling pathways
Source: Front Microbiol. 2025 Sep 25;16:1676456. doi: 10.3389/fmicb.2025.1676456 (PMC12507761; doi:10.3389/fmicb.2025.1676456)

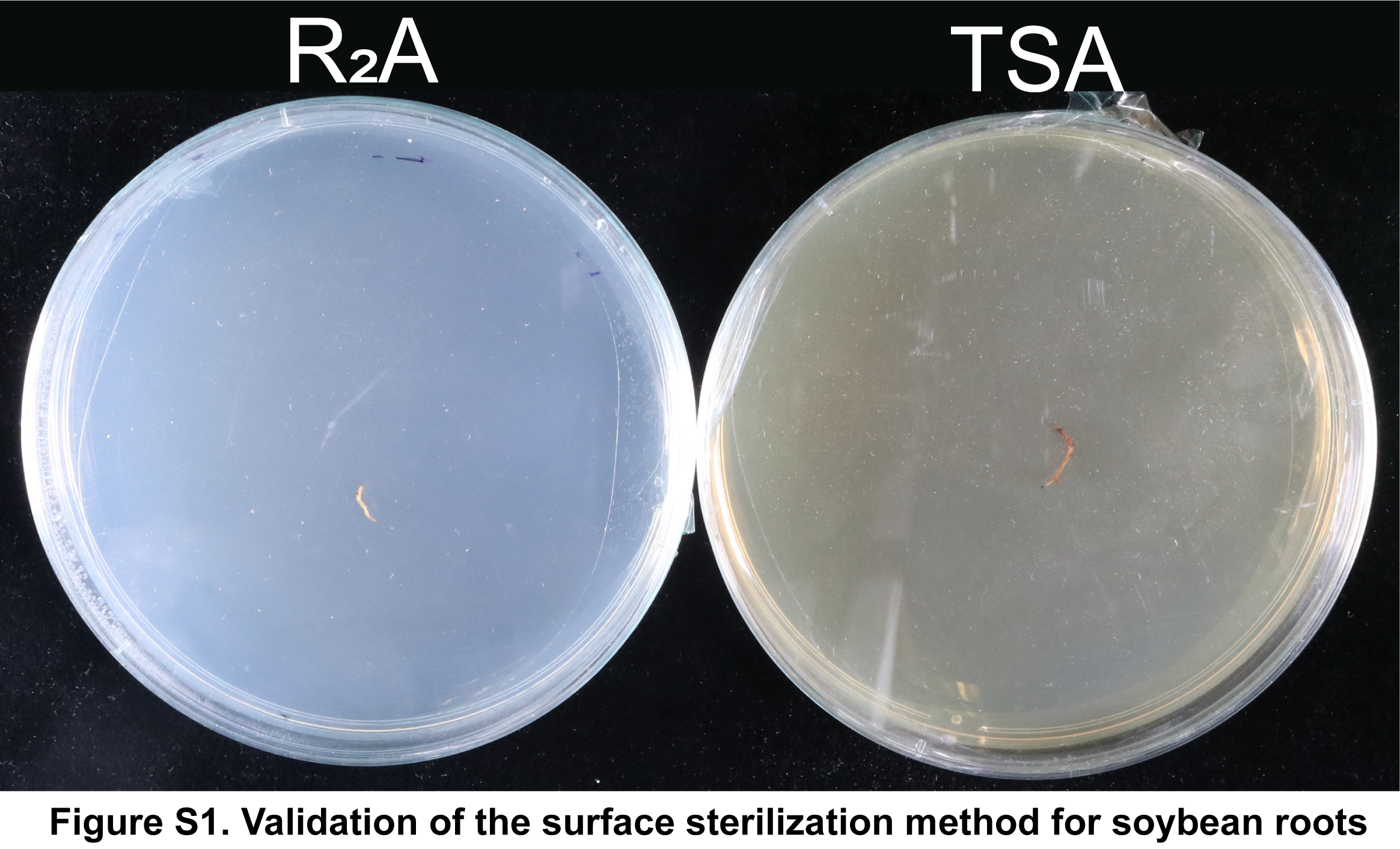

Supplement: Supplementary file 1 [file Image_1.TIF]

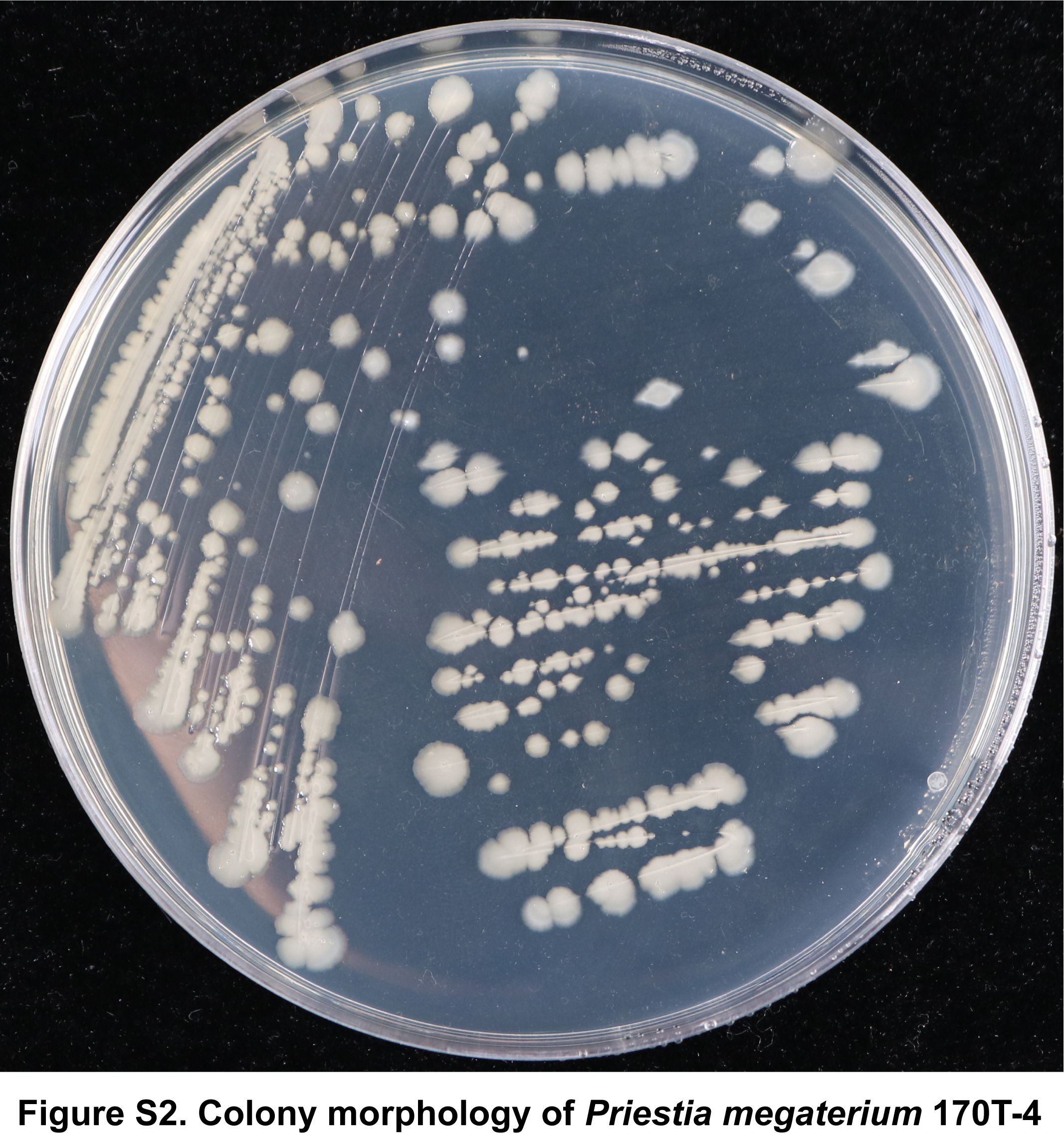

Supplement: Supplementary file 2 [file Image_2.TIF]

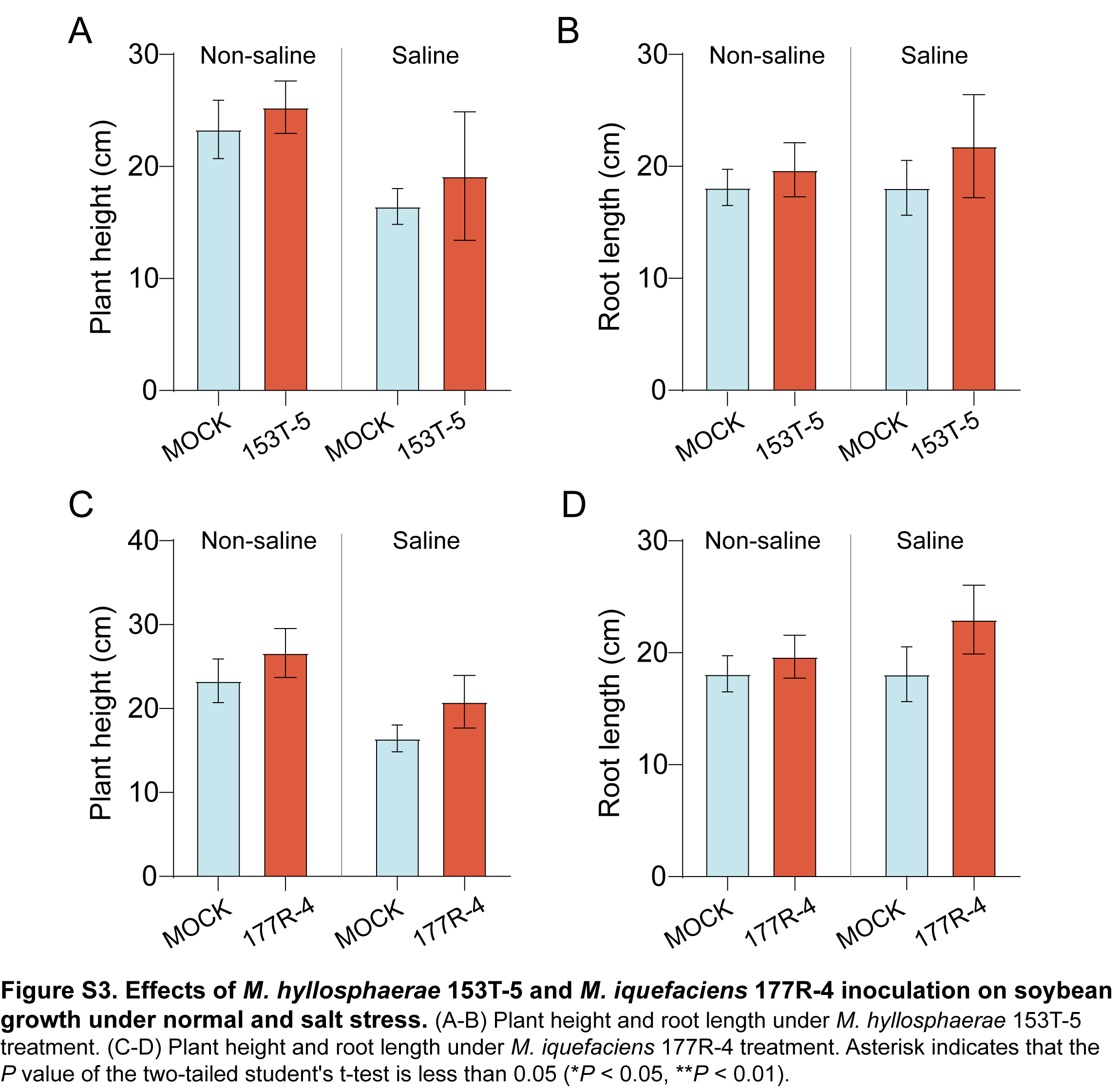

Supplement: Supplementary file 3 [file Image_3.TIF]
